# Uma Nova Era na Avaliação da Hipertensão: Análise Não Invasiva da Pressão Central e das Ondas Intracranianas

**DOI:** 10.36660/abc.20250277

**Published:** 2025-05-28

**Authors:** Juan Carlos Yugar-Toledo, Heitor Moreno

**Affiliations:** 1 Faculdade de Medicina de São José do Rio Preto SP Brasil Faculdade de Medicina de São José do Rio Preto, São José do Rio Preto, SP – Brasil; 2 Instituto de Cardiologia e Endocrinologia Rio Preto Ltda São Paulo SP Brasil Instituto de Cardiologia e Endocrinologia Rio Preto Ltda, São José do Rio Preto, São Paulo, SP – Brasil; 3 Universidade Estadual de Campinas Campinas SP Brasil Universidade Estadual de Campinas, Campinas, SP – Brasil

**Keywords:** Hipertensão, Pressão Arterial, Forma de Onda Intracraniana, Circulação Cerebrovascular

A relação entre hipertensão arterial e fluxo sanguíneo cerebral (FSC) tem sido extensivamente estudada devido ao papel que a pressão arterial (PA) desempenha na manutenção de uma perfusão cerebral adequada. A autorregulação cerebral é o mecanismo pelo qual o FSC permanece relativamente constante, apesar das variações na pressão de perfusão. Em indivíduos normotensos, a faixa de autorregulação situa-se aproximadamente entre pressões arteriais médias de 60 a 150 mmHg.^1 , 2^

Em pacientes hipertensos, há evidências de que essa curva se desloca para valores mais altos, fazendo com que: a) O cérebro hipertenso tolere pressões sanguíneas mais altas antes que ocorra vasodilatação ou vasoconstrição; b) Por outro lado, quedas abruptas da PA podem resultar em hipoperfusão cerebral, uma vez que o limiar inferior para autorregulação pode ser maior.

A hipertensão crônica desencadeia uma série de alterações estruturais e funcionais nos vasos cerebrais que comprometem o fluxo sanguíneo adequado. A remodelação vascular é uma característica marcante: artérias e arteríolas cerebrais sob alta pressão sofrem espessamento da parede muscular e estreitamento do lúmen, levando a um aumento na relação parede/lúmen dos vasos, reduzindo a complacência vascular e dificultando a regulação do fluxo. Essa remodelação "para dentro" reduz o diâmetro interno dos vasos e aumenta a resistência cerebrovascular, o que pode limitar o fluxo, especialmente quando a perfusão é crítica (por exemplo, durante episódios isquêmicos).^3^

Estudos indicam que essa remodelação é impulsionada por fatores neuro-humorais elevados na hipertensão, incluindo a ativação do sistema renina-angiotensina-aldosterona e o estresse oxidativo elevado, que promove a proliferação de células vasculares e a deposição de matriz.^4^ Além disso, modelos experimentais de hipertensão demonstraram rarefação microvascular, ou seja, redução na densidade de arteríolas e capilares cerebrais. Em modelos animais hipertensos, observou-se uma redução de até 25% a 50% no número de pequenas artérias piais e capilares intracerebrais, o que reduz a rede de perfusão disponível.^5^ Do ponto de vista funcional, a hipertensão também causa disfunção endotelial e alteração da reatividade vascular. Em condições normais, o endotélio libera fatores vasodilatadores e vasoconstritores, como o óxido nítrico, para ajustar o calibre arterial de acordo com as necessidades metabólicas. Na hipertensão crônica, a biodisponibilidade do óxido nítrico é reduzida e há um desequilíbrio em outras vias vasodilatadoras, resultando em uma resposta vasodilatadora prejudicada.^6 , 7^ As abordagens sobre a relação entre hipertensão e FSC têm se beneficiado dos avanços nas técnicas de imagem e métodos de avaliação funcional, como a ressonância magnética funcional para analisar a perfusão cerebral (ASL – Arterial Spin Labeling) e a reatividade cerebral a estímulos;^8^ o doppler transcraniano: que permite avaliar a velocidade do fluxo nas artérias cerebrais e estimar a autorregulação dinâmica;^9^ e a tomografia por emissão de pósitrons: que pode quantificar o metabolismo e a perfusão cerebral com maior precisão, mas é mais custosa e menos disponível. Esses métodos ajudam a identificar alterações hemodinâmicas e estruturais decorrentes da hipertensão em estágios iniciais, permitindo intervenções preventivas mais eficazes.

Entretanto, um fenômeno bastante estudado, mas pouco valorizado na prática clínica, é a forma de onda da pressão intracraniana (PIC).^10^ Variações na PIC foram demonstradas de acordo com alterações no volume e pressão intracranianos.^11 , 12^ Recentemente, foi desenvolvida uma técnica não invasiva que avalia deformações micrométricas do crânio ao longo do ciclo cardíaco e é capaz de reproduzir a PIC, com forte correlação com a morfologia invasiva da forma de onda da PIC.^13^ A correlação entre os valores da PIC média e a relação P2/P1 é linear de acordo com estudos publicados por Moraes et al.,^12^ e Brasil et al.^11^

No trabalho, intitulado " Avaliação Não Invasiva da Pressão Arterial Central e da Forma de Onda Intracraniana em Pacientes Hipertensos: Um Estudo Transversal",^14^ os autores estudaram pacientes hipertensos utilizando um novo método de mensuração não invasiva da pressão intracraniana. Esse método foi realizado com os pacientes em decúbito dorsal e monitorados por sete minutos. Utilizando a avaliação não invasiva b4c, o ponto de corte identificado para definir a hipertensão intracraniana (HTIC) pela razão P2/P1 foi ≥ 1,2, enquanto o ponto de corte para tempo para o pico (TPP) foi ≥ 0,25 segundos. Os valores de P2/P1 de 1,0 a 1,19 e os valores de TPP de 0,20 a 0,24 segundos foram considerados zona cinzenta de complacência intracraniana anormal, mas não HTIC.

Foi realizada uma análise para estudar a associação entre HTIC e valores centrais de PA e velocidade de onda de pulso. Valores mais elevados de cPAS (pressão arterial sistólica), cPAD (pressão arterial diastólica) e pPAD foram encontrados em pacientes com HTIC, avaliados pelo critério da razão P2/P1. Ao definir o HTIC por TPP ≥ 0,25 segundos, tanto a PAD central quanto a periférica foram maiores nesses pacientes. Neste estudo, os autores encontraram uma mediana da razão P2/P1 de 1,4 (1,2-1,5) e um valor mediano de TPP de 0,24 (0,21-0,29) segundos na população analisada.

Os autores não encontraram diferenças nos valores médios da razão P2/P1 e TPP nas variáveis sociodemográficas, antropométricas e clínicas registradas, exceto pela diferença feminina observada quanto a um maior TPP na população feminina. Um ponto importante é que a incidência de acidente vascular cerebral e demência é maior entre as mulheres, e os achados de maior TPP neste estudo, bem como a maior razão P2/P1, apoiam a hipótese de que a capacidade autorregulatória da pressão intracraniana e a permeabilidade da barreira hematoencefálica são afetadas de forma diferente em homens e mulheres.

Por fim, na análise considerando os pontos de corte de 1,2 e 0,25 seg para P2/P1 e TPP, os autores encontraram significância estatística da cPAS, cPAD e pPAD na detecção dessas diferenças em relação a P2/P1; A cPAD e a pPAD foram capazes de detectar essas diferenças em relação ao TPP, e os mesmos parâmetros mantiveram uma correlação moderada e significativa entre as medidas de PA e as variáveis da PIC.

A implementação dessas tecnologias pode transformar a prática clínica, tornando-a mais acessível e menos desconfortável para os pacientes. No entanto, desafios como custo e treinamento especializado ainda precisam ser superados para que essas ferramentas sejam amplamente adotadas. O futuro da medicina parece promissor com avanços como esse, que priorizam a segurança e o bem-estar do paciente.
