# Supplementary material for: Uma Nova Era na Avaliação da Hipertensão: Análise Não Invasiva da Pressão Central e das Ondas Intracranianas
Source: Arq Bras Cardiol. 2025 May 28;(5):e20250277. [Article in Portuguese] doi: 10.36660/abc.20250277 (PMC12212381; doi:10.36660/abc.20250277)
Supplement: Translation

# Translation: A New Era of Hypertension Assessment: Non-Invasive Central and Intracranial Waveform Analysis

**DOI:** 10.36660/abc.20250277i

**Authors:** Juan Carlos Yugar-Toledo, Heitor Moreno

**Affiliations:** 1 SP Brazil Faculdade de Medicina de São José do Rio Preto, São José do Rio Preto, SP – Brazil; 2 São Paulo SP Brazil Instituto de Cardiologia e Endocrinologia Rio Preto Ltda, São José do Rio Preto, São Paulo, SP – Brazil; 3 Campinas SP Brazil Universidade Estadual de Campinas, Campinas, SP – Brazil

**Keywords:** Hipertensão, Pressão Arterial, Forma de Onda Intracraniana, Circulação Cerebrovascular

The relationship between arterial hypertension and cerebral blood flow (CBF) has been extensively studied due to the role that blood pressure plays in maintaining adequate cerebral perfusion. Cerebral autoregulation is the mechanism by which cerebral blood flow remains relatively constant despite variations in perfusion pressure. In normotensive individuals, the autoregulation range is approximately between mean arterial pressures of 60 to 150 mmHg.^1 , 2^

In hypertensive patients, there is evidence that this curve shifts to higher values, causing: a) The hypertensive brain to tolerate higher blood pressures before vasodilation or vasoconstriction occurs; b) On the other hand, abrupt drops in blood pressure can result in cerebral hypoperfusion, since the lower threshold for autoregulation may be higher.

Chronic hypertension triggers a series of structural and functional changes in cerebral vessels that compromise adequate blood flow. Vascular remodeling is a striking feature: cerebral arteries and arterioles under high pressure undergo thickening of the muscular wall and narrowing of the lumen, leading to an increase in the wall/lumen ratio of the vessels, reducing vascular compliance and making flow regulation difficult. This "inward" remodeling reduces the internal diameter of the vessels and increases cerebrovascular resistance, which can limit flow, especially when perfusion is critical (for example, during ischemic episodes).^3^

Studies indicate that this remodelling is driven by elevated neurohumoral factors in hypertension, including activation of the renin-angiotensin-aldosterone system (RAAS) and elevated oxidative stress, which promotes vascular cell proliferation and matrix deposition.^4^In addition, experimental models of hypertension have shown microvascular rarefaction – that is, a reduction in the density of cerebral arterioles and capillaries. In hypertensive animal models, up to 25–50% decrease in the number of small pial arteries and intracerebral capillaries has been observed, which reduces the available perfusion network.^5^From a functional point of view, hypertension also causes endothelial dysfunction and altered vascular reactivity. Under normal conditions, the endothelium releases both vasodilatory and vasoconstrictor factors, such as nitric oxide, to adjust arterial caliber according to metabolic needs. In chronic hypertension, nitric oxide bioavailability is reduced, and there is an imbalance in other vasodilatory pathways, resulting in an impaired vasodilatory response.^6 , 7^The approaches to the relationship between hypertension and cerebral blood flow have benefited from advances in imaging techniques and functional assessment methods, such as Functional Magnetic Resonance Imaging (fMRI) to analyze cerebral perfusion (ASL – Arterial Spin Labeling) and brain reactivity to stimuli;^8^Transcranial Doppler (TCD): which makes it possible to assess flow velocity in cerebral arteries and estimate dynamic autoregulation;^9^and PET (Positron Emission Tomography): which can quantify cerebral metabolism and perfusion with greater precision, but is more costly and less available. These methods help to identify hemodynamic and structural changes resulting from hypertension in the early stages, allowing more effective preventive interventions.

However, a well-studied phenomenon but little valued in clinical practice is the intracranial pressure (ICP) waveform.^10^Variations in ICP have been demonstrated according to changes in intracranial volume and pressure.^11 , 12^Recently, a non-invasive technique was developed that evaluates micrometric deformations of the skull throughout the cardiac cycle and is capable of reproducing ICP, with a strong correlation with the invasive morphology of the ICP waveform.^13^The correlation between mean ICP values and the P2/P1 ratio is linear according to studies published by Moraes et al.,^12^and Brasil et al.^11^

In the work entitled "Non-Invasive Central Blood Pressure and Intracranial Waveform Assessment in Hypertensive Patients: A Cross-Sectional Study" the authors studied hypertensive patients using a new method for non-invasive intracranial pressure measurement. This method was performed with patients in a lying position and monitored for seven minutes. Using the b4c non-invasive evaluation, the cut-off point identified to define intracranial hypertension (ICHT) by the P2/P1 ratio was ≥ 1.2, while the cut-off for TTP was ≥ 0.25 seconds. The values of P2/P1 from 1.0 to 1.19 and the TTP values from 0.20 to 0.24 seconds were considered a grey zone of abnormal intracranial compliance but not ICHT.

Analysis was performed to study the association between ICHT and central BP and PWV values. Higher cSBP, cDBP, and pDBP were found in patients with ICHT assessed by the P2/P1 ratio values criterion. When ICHT was defined by TTP ≥ 0.25 seconds, both central and peripheral DBP were higher in these patients. In this study, the authors found a median P2/P1 ratio of 1.4 (1.2 – 1.5) and a median TTP value of 0.24 (0.21 – 0.29) seconds in the analyzed population.

The authors found no differences in the mean values of the P2/P1 ratio and TTP in the recorded sociodemographic, anthropometric, and clinical variables except for the female difference observed regarding a greater TTP in the female population. An important point is that the incidence of stroke and dementia is higher among females, and the findings of greater TTP in this study, as well as the greater P2/P1 ratio, support the hypothesis that the autoregulatory capacity of intracranial pressure and BBB permeability are differently affected in males and females.

Finally, in the analysis considering the cut-offs of 1.2 and 0.25 sec for P2/P1 and TTP, the authors found the statistical significance of cSBP, cDBP, and pDBP in detecting these differences concerning P2/P1; cDBP and pDBP were able to detect these differences concerning TTP, and the same parameters maintained a moderate and significant correlation between the BP measurements and ICP variables.

The implementation of these technologies can transform clinical practice, making it more accessible and less uncomfortable for patients. However, challenges such as cost and specialized training still need to be overcome for these tools to be widely adopted. The future of medicine looks promising with advances like this, which prioritize patient safety and well-being.
